# Validation of Infrared Thermal Imaging for Grading of Cellulite Severity: Correlation with Clinical and Anthropometric Assessments

**DOI:** 10.3390/jcm15020913

**Published:** 2026-01-22

**Authors:** Patrycja Szczepańska-Ciszewska, Andrzej Śliwczyński, Bartosz Mruk, Wojciech Michał Glinkowski, Patryk Wicher, Adam Sulimski, Anna Wicher

**Affiliations:** 1 National Medical Institute of the Ministry of the Interior and Administration, 02-507 Warsaw, Poland; 2 Center of Excellence “TeleOrto” for Telediagnostics and Treatment of Disorders and Injuries of the Locomotor System, Department of Medical Informatics and Telemedicine, Medical University of Warsaw, 02-091 Warsaw, Poland; 3 Higher School of Business—National Louis University Based in Nowy Sącz, 33-300 Nowy Sącz, Poland

**Keywords:** cellulite, infrared thermography, thermographic scale, noninvasive diagnosis, objective assessment, skin temperature mapping, severity grading, microcirculation, anthropometric correlation, aesthetic medicine, psychology in medicine, management in medicine

## Abstract

**Background/Objectives:** Cellulite is a common aesthetic condition in women, traditionally assessed using visual inspection and palpation-based scales that are inherently subjective. Therefore, image-based methods that may support standardized severity grading are of growing interest. To evaluate infrared thermography as an imaging-based method for grading cellulite severity and to perform methodological validation of a newly developed thermographic classification scale by comparing it with clinical palpation and anthropometric parameters. **Methods:** This retrospective, non-interventional study analyzed anonymized clinical and thermographic data from 81 women with clinically assessed cellulite. Cellulite severity was evaluated using the Nürnberger–Müller palpation scale and a newly developed five-point thermographic scale based on skin surface temperature differentials and histogram pattern analysis. The associations between the assessment methods were evaluated using ordinal statistical measures, and agreement was assessed using weighted Cohen’s kappa statistics. **Results:** Thermographic grading demonstrated high agreement with palpation-based assessment, with a percentage agreement of 93.8% and an almost perfect agreement based on weighted Cohen’s κ. A strong ordinal association was observed between the methods. Thermography consistently classified a subset of cases as one grade higher compared with palpation. No statistically significant associations were observed between thermographic grade and body mass index or waist-to-hip ratio. **Conclusions:** Infrared thermography enables image-based grading of cellulite severity and shows a strong concordance with established palpation scales. The proposed thermographic classification provides preliminary methodological validation of an imaging-based grading approach. Further multicenter studies involving multiple assessors and diverse populations are required to assess reproducibility, specificity, and potential clinical applicability.

## 1. Introduction

Cellulite, clinically referred to as edematous fibrosclerotic panniculopathy, is a prevalent connective tissue disorder and an aesthetic concern that affects up to 85% of postpubertal women across various ethnicities. Clinically, it is characterized by dermal dimpling, nodularity, and a distinctive “orange peel” skin appearance, predominantly observed on the thighs, buttocks, and hip regions [1,2]. Cellulite, linked to the biological characteristics of adipose tissue, is characterized by changes in microcirculation, adipose tissue, and connective tissue tension, which, in turn, affect skin temperature, as detected by thermography [3,4]. In the case of cellulite, adipocyte hypertrophy and fibrotic changes impede circulation, causing temperature differences between ischemic areas, which are cooler, and congested areas, which are warmer. These differences give rise to thermographic patterns associated with varying degrees of cellulite severities. In clinical practice, the severity of cellulite is assessed by visual inspection and palpation using scales such as the Nürnberger-Müller classification [5,6,7,8]. Conventional approaches to evaluating cellulite, which rely on visual inspection and palpation, are inherently subjective and operator-dependent. This results in variability among observers under different conditions, underscoring the need for more reliable diagnostic methods. Infrared thermography is a noninvasive technique for assessing skin surface temperature, which reflects the underlying perfusion, tissue structure, and microcirculatory processes [9,10,11]. Studies have shown that thermography can distinguish between healthy and affected tissues; however, standardized diagnostic scales for cellulite remain underexplored [12,13,14]. The primary objective of this study was the methodological validation of an objective thermographic grading scale, rather than demonstrating its superiority in clinical outcomes.

## 2. Materials and Methods

### 2.1. Study Design and Population

This retrospective, cross-sectional study analyzed anonymized routine medical and cosmetology records from Studio AVENIR—Medicine and Aesthetic Cosmetology (Warsaw, Poland) collected from December 2023 to 15 December 2024. The study included 81 Caucasian women (aged 22–72 years) who presented for clinical evaluation of suspected cellulite.

This study was conducted in accordance with the principles of the Declaration of Helsinki. This study was conducted as a retrospective, non-interventional analysis of anonymized clinical and thermographic data. Following the peer-review process and at the request of the reviewers and the Editorial Office, the study documentation was submitted to a Bioethics Committee, which issued formal approval confirming that the study met ethical standards and did not require additional patient consent.

All participants were adults who volunteered to participate in this study. The participants received information about the procedures and provided electronic informed consent for clinical assessment, thermographic imaging, and the use of anonymized data for research. The participants were informed that their participation in the study was voluntary and that they could withdraw at any time without consequences. No personal identifiable information was collected, and all data were anonymized. Data storage and processing complied with the applicable data protection regulations, and access to the data was restricted to the research team. The participants received no financial or other compensation.

### 2.2. Inclusion and Exclusion Criteria

The inclusion criterion was women presenting with suspected cellulite, as confirmed by specialist clinical evaluation. The exclusion criteria were pregnancy or breastfeeding, uncontrolled systemic disease, medications affecting the skin or circulation, prior cellulite treatment, active cancer therapy, varicose veins, and contraindications for thermographic assessment.

### 2.3. Anthropometric Assessment

Height, weight, waist circumference, and hip circumference were measured according to standard protocols. Body Mass Index (BMI) was calculated as weight (kg)/height^2^ (m^2^) and classified according to the World Health Organization (WHO) criteria: <18.5, underweight; 18.5–24.9, normal; 25.0–29.9, preobesity; 30.0–34.9, obesity I; 35.0–39.9, obesity II; and ≥40, obesity III. The waist-to-hip ratio (WHR) was derived from the waist-to-hip circumference. WHR > 0.85 in women, indicated an android (“apple”) fat distribution; WHR ≤ 0.85 indicated gynoid (“pear”) fat distribution [15]. These parameters were used to categorize body type and characterize anthropometric status. Cellulite severity was visually graded and assessed by manual palpation using the simplified Nürnberger–Müller scale (Table 1).

The clinical assessment was performed by trained specialists with the participants in the standing and supine positions, with relaxed muscles and during voluntary contraction.

### 2.4. Thermographic Assessment

Infrared imaging was performed with an FLIR P620 camera (Teledyne FLIR, Lucca, Italy, 640 × 480 px, NETD ≤ 40 mK, spectral range 7.5–13 µm, emissivity 0.98) under the European Thermological Society standards.

The imaging was conducted in a controlled environment (constant room temperature, controlled humidity, and stable lighting). The participants were acclimatized for 20 min before imaging and refrained from eating for two h before the examination. The participants avoided wearing tight clothing and engaging in recent physical exertion.

Acquisition: Four views (anterior, posterior, left, and right lateral) of the thighs and buttocks with relaxed muscles were acquired, and the process was repeated with tensed muscles.

Analysis: FLIR Tools software ver. 6.4.18039.1003 Teledyne FLIR LLC, “rainbow HC” palette; temperature range 26.6–36.6 °C; reflected temperature 20 °C.

### 2.5. Novel Thermographic Cellulite Scale

A five-grade ordinal scale (0–IV) was developed based on the maximum–minimum skin surface temperature difference (ΔT) and color histogram patterns (Table 2). The thermographic cellulite scale assigns grades 0–IV based on the following criteria: measurement of the maximum and minimum skin surface temperatures, assessment of histogram color patterns, and grading based on specific temperature differentials and pattern appearance.

All thermographic images were graded by a single experienced assessor blinded to the palpation results (Supplementary Table S1). Given the strong concordance between the thermographic and clinical palpation scales, we explored how these objective grades relate to standard anthropometric measures, such as BMI and WHR.

### 2.6. Statistical Analysis

Descriptive statistics were calculated for age, BMI, WHR, and cellulite severity. As both the thermographic and palpation-based cellulite grades were ordinal variables, the ordinal association between the methods was assessed using Spearman’s rank correlation coefficient (ρ) as the primary measure, with Kendall’s τ-b reported as a robustness check to account for tied ranks. The agreement between thermographic and palpation-based grading was quantified using percent agreement and weighted Cohen’s κ with quadratic weights along with 95% confidence intervals (CIs).

No formal correction for multiple comparisons was performed. The analyses were limited to a small number of predefined hypothesis-driven comparisons, and the study was intended for methodological validation rather than confirmatory hypothesis testing. Therefore, the results should be interpreted as exploratory.

Thermographic grading was performed by a single experienced assessor blinded to the palpation results; therefore, the inter-rater reliability could not be calculated. This limitation has been acknowledged. All tests were two-sided, with a significance level of α = 0.05. Statistical analyses were performed using the SPSS Statistics software (version 29.0; IBM Corp., Armonk, NY, USA).

#### Sample Size Justification

This was a retrospective, exploratory study. No a priori sample size or power calculation was performed because the analysis was based on all eligible cases available during the study period. Therefore, the sample size was determined by data availability rather than hypothesis-driven power estimation.

## 3. Results

This study included 81 women (mean age 37.45 years, SD 8.27; mean BMI 23.01, SD 4.19). The grade distributions for thermography and palpation (relaxed muscles) are shown in Table 3. Complete agreement was observed for grades 0–II. All five cases classified as grade IV by thermography were classified as grade III by palpation.

### 3.1. Agreement Between Methods

The agreement matrix for the assessments performed on relaxed muscles is presented in Table 4.

Values represent number of cases. Complete agreement was observed for grades 0–II. All grade IV cases identified by thermography were classified as grade III by palpation.

The agreement statistics demonstrated a percentage agreement of 93.8% (76/81). Cohen’s κ was 0.92 (95% CI 0.84–1.00), and weighted κ (quadratic weights) was 0.95. A perfect ordinal association between thermographic and palpation-based grading was observed (Spearman’s ρ = 1.00, *p* < 0.001), which was confirmed by Kendall’s τ-b (τ-b = 1.00, *p* < 0.001). In no case was palpation assigned a grade higher than thermography. All discrepancies occurred in a single direction without rank reversals.

### 3.2. Muscle Tension Effect

No statistically significant differences in grade assignment were observed between relaxed and tensed muscle assessments using either method.

### 3.3. Temperature Differential by Grade (Thermography)

The mean ΔT increased stepwise from 0.9 °C (grade 0) to >5 °C (grade IV), with limited overlap between adjacent grades (Table 5).

### 3.4. Anthropometric Correlations

No statistically significant association was observed between BMI and thermographic grade (Spearman’s ρ = 0.16, *p* = 0.16). Similarly, no significant association was found between WHR and thermographic grade (Spearman’s ρ = 0.00, *p* = 0.99). Based on WHR classification, 50 participants (61.7%) exhibited an android (“apple”) body fat distribution, while 31 participants (38.3%) exhibited a gynoid (“pear”) distribution.

## 4. Discussion

This study evaluated the accuracy of infrared thermography as an objective method for classifying cellulite severity by comparing a standard thermographic scale with a recognized palpation-based assessment method. The results showed a high level of agreement between thermographic and clinical assessments, with a percentage agreement of 93.8% and a weighted Cohen’s κ coefficient of 0.95, indicating substantial agreement between the methods. Notably, the perfect ordinal relationship observed in this study (Spearman’s ρ = 1.00; Kendall’s τ-b = 1.00) should be interpreted as evidence of a strictly monotonic relationship between thermographic and palpation-based grading, rather than exact categorical equivalence. All discrepancies occurred in a single direction, without rank reversals, indicating systematic differences at higher severity grades rather than random disagreement. Importantly, the high level of agreement observed in this study should be interpreted in the context of the controlled assessment conditions. Both thermographic and palpation-based grading were performed by a single experienced assessor under standardized settings. Therefore, the observed agreement does not negate the well-documented operator dependence of palpation-based cellulite assessment in routine clinical practice. Instead, it reflects methodological concordance under controlled conditions and highlights the potential value of thermography as a standardized documentation tool rather than evidence of operator independence in real-world settings. In routine clinical settings involving multiple examiners and less standardized conditions, variability of palpation-based grading would be expected to be substantially higher.

In this study, all cases classified as grade IV by thermography were systematically assigned grade III during palpation-based assessment. This finding reflects a consistent methodological difference between temperature-based imaging and tactile clinical examination rather than random disagreement. Importantly, in the absence of an independent reference standard, such as histopathological correlation or outcome-based validation, the clinical significance of this difference cannot be determined. Therefore, this systematic shift in grading should be interpreted as methodological discordance between two assessment approaches operating on different underlying principles, rather than evidence of diagnostic superiority or underestimation by either method. The physiological basis for thermographic assessment lies in the relationship between microvascular function, adipose tissue structure, and heat transfer [1,6,16]. Fibrotic remodeling and adipocyte hypertrophy can impair perfusion, leading to cooler areas of ischemia, whereas local hyperemia or inflammatory processes can cause warmer regions [17,18,19,20,21,22]. These changes result in heterogeneous thermal patterns that correspond to the cellulite severity [2,23]. The characteristic thermographic morphologies observed in higher grades, such as patchy low-temperature areas and marked thermal heterogeneity, are consistent with the pathophysiological mechanisms described in histological studies and vascular imaging [5,16,24].

A key contribution of this study is the application and validation of a five-grade thermographic scale based on clearly defined skin temperature differences and histogram-based pattern analysis. Unlike previous approaches based on descriptive assessments or proprietary indices, this method provides transparent quantitative thresholds that facilitate repeatability and comparability across assessments [25,26,27]. The use of standardized environmental conditions, controlled acclimatization, and muscle condition assessment further strengthens the internal consistency of the protocol and reduces the sources of measurement variability [7,28,29,30].

Contrary to common assumptions [16,31,32], no statistically significant relationship was observed between thermographic grade and BMI or WHR in this group. These results confirm the multifactorial nature of cellulite, suggesting that the severity of the condition cannot be explained solely by overall obesity or regional fat distribution [5,7,33]. Structural changes in connective tissue, impaired microcirculation, and tissue remodeling may play a more significant role than anthropometric measurements alone, which is consistent with previous reports emphasizing the complex pathophysiology of cellulite [31,34].

An additional finding of the present study was the absence of statistically significant differences in grading between relaxed and tensed muscle conditions. Although muscle tension might be expected to influence surface morphology and palpation-based assessment, this effect was not observed under the standardized imaging and examination conditions applied in this study. All assessments were performed in a controlled environment with fixed positioning, predefined muscle activation instructions, and consistent acquisition protocols, which may have minimized posture- and tension-related variability. This finding should therefore be interpreted as context-specific and methodological, rather than evidence that muscle tension has no effect on cellulite appearance in routine clinical practice.

This study had several limitations. Given the retrospective nature of the study, single-center setting, homogeneous study population, and thermographic assessment performed by a single evaluator, these results should be considered preliminary and hypothesis generating. Thermographic classification was performed by a single experienced rater, which prevented assessment of inter-rater agreement. Within these limitations, thermography may be considered a complementary documentation tool rather than a diagnostic replacement for clinical examination.

The study did not include histopathological reference standards or clinical outcome measures; therefore, the clinical relevance of higher thermographic grades cannot be established, and no claims regarding diagnostic superiority can be made. Furthermore, the lack of a cellulite-free control group limits the evaluation of diagnostic specificity. Device calibration and the use of static rather than dynamic imaging may also affect the thermal measurements, although these factors are consistent with the methodologies used in comparable studies. The absence of a priori power calculation and correction for multiple tests reflects the exploratory and retrospective design of the study and limits the inferential scope of the findings.

Despite these limitations, the results indicate that infrared thermography provides an orderly, quantitative complement to the assessment of cellulite severity based on palpation alone. Its non-invasive nature and reliance on imaging enable standardized documentation and may improve the detection of advanced structural changes that are less visible on clinical examinations. Future studies should focus on prospective multicenter validation, inclusion of diverse populations and control groups, and direct comparisons with other imaging methods, such as ultrasonography or magnetic resonance imaging. Longitudinal studies are required to determine the usefulness of thermography as an outcome measure for monitoring therapeutic interventions.

The validated thermographic classification method is a promising tool for objectively classifying cellulite severity. With further validation, infrared thermography may contribute to more standardized documentation, increase comparability between studies, and contribute to improved clinical and research applications in aesthetic medicine.

The present study did not evaluate cost-effectiveness, patient-reported outcomes, or clinical utility. Assessment of economic value and patient-centered benefit of thermographic cellulite grading would require dedicated prospective studies incorporating health-economic analyses and outcome-based endpoints.

## 5. Conclusions

This study provides methodological validation of a five-grade thermographic classification system for documenting cellulite severity based on objective skin surface temperature patterns. Under controlled conditions, infrared thermography demonstrated high agreement with palpation-based assessment, reflecting methodological concordance rather than diagnostic superiority. The observed differences between thermographic and palpation-based grading, particularly at higher severity levels, should be interpreted as methodological discordance between two assessment approaches operating on different underlying principles. In the absence of histopathological correlation, outcome-based validation, or prospective assessment, no conclusions regarding clinical superiority, diagnostic performance, or clinical implementation can be drawn. Accordingly, infrared thermography should be considered an image-based documentation and research tool for standardized grading of cellulite severity rather than a replacement for clinical examination. Further prospective, multicenter studies incorporating independent reference standards, clinical outcomes, and multiple assessors are required before any clinical or research endpoint applications can be considered.

## Figures and Tables

**Table 1:** Cellulite severity scoring, both visually and by manual palpation, was performed using the simplified Nürnberger–Müller scale.

Grade	Criteria
0	No visible changes
I	Smooth skin at rest; dimpling only when pinched or on muscle contraction
II	“Orange peel” visible standing; resolves lying
III	“Orange peel” is visible in all positions at rest.

**Table 2:** The developed scale (0–IV) showing the skin surface temperature differences (ΔT) and color histogram patterns.

Grade	ΔT (°C)	Description
0	0.5–1.9	Uniform thermal distribution
I	2.0–2.9	Minor irregularity, mild blue/green/yellow dots
II	3.0–3.9	Moderate heterogeneity, more dots, blue dominant
III	4.0–4.9	“Leopard skin” pattern, red/yellow patches
IV	≥5.0	Marked thermal heterogeneity with large low-temperature (ischemic) areas, low-temperature voids (“black holes”), intense contrast.

**Table 3:** Cellulite grade distribution by method.

Grade	Thermography n (%)	Palpation n (%)
0	1 (1.2)	1 (1.2)
I	12 (14.8)	12 (14.8)
II	28 (34.6)	28 (34.6)
III	35 (43.2)	40 (49.4)
IV	5 (6.2)	0 (0.0)
Total	81 (100)	81 (100)

**Table 4:** Cross-tabulation of grades (thermography vs. palpation).

Thermography/Palpation	0	1	2	3	4
0	1	0	0	0	0
1	0	12	0	0	0
2	0	0	28	0	0
3	0	0	0	35	0
4	0	0	0	5	0

**Table 5:** ΔT ranges for each thermographic grade.

Grade	ΔT Range (°C)
0	0.5–1.9
I	2.0–2.9
II	3.0–3.9
III	4.0–4.9
IV	5.0–5.9

## Data Availability

The raw data supporting the conclusions of this article will be made available by the authors on request.

## Supplementary Materials

The following supporting information can be downloaded at https://www.mdpi.com/article/10.3390/jcm15020913/s1, Table S1: The table presents the Thermographic Cellulite Severity Rating Scale.

## References

[1] Eber A.E., Hooper P.B., Labadie J.G., Kandula P., Dover J., Kaminer M.S. (2023). Cellulite Management Update. Adv. Cosmet. Surg..

[2] Tokarska K., Tokarski S., Woźniacka A., Sysa-Jędrzejowska A., Bogaczewicz J. (2018). Cellulite: A cosmetic or systemic issue? Contemporary views on the etiopathogenesis of cellulite. Postep. Dermatol. Alergol..

[3] Bass L.S., Kaminer M.S. (2020). Insights Into the Pathophysiology of Cellulite: A Review. Dermatol. Surg..

[4] Ribeiro do Vale Silva J., Rodrigues de Araújo M.d.G., Guerino M.R. (2022). The use of a thermographic camera in aiding the diagnosis of cellulite appearance: 10.15343/0104-7809.202246279288. O Mundo Da Saúde.

[5] Young V.L., DiBernardo B.E. (2021). Comparison of Cellulite Severity Scales and Imaging Methods. Aesthet. Surg. J..

[6] Bauer J., Grabarek M., Migasiewicz A., Podbielska H. (2018). Non-contact thermal imaging as potential tool for personalized diagnosis and prevention of cellulite. J. Therm. Anal. Calorim..

[7] Gabriel A., Chan V., Caldarella M., Wayne T., O’Rorke E. (2023). Cellulite: Current Understanding and Treatment. Aesthet. Surg. J. Open Forum.

[8] Szczepańska P., Borzuchowska M., Strzelec A., Marczak M., Kozłowski R. (2024). Diagnostic scales and methods for assessing the severity of gynoid lipodystophy. J. Health Policy Outcomes Res..

[9] Ramirez-GarciaLuna J.L., Bartlett R., Arriaga-Caballero J.E., Fraser R.D.J., Saiko G. (2022). Infrared Thermography in Wound Care, Surgery, and Sports Medicine: A Review. Front. Physiol..

[10] Straburzyńska-Lupa A., Korman P., Śliwicka E., Kryściak J., Ogurkowska M.B. (2022). The use of thermal imaging for monitoring the training progress of professional male sweep rowers. Sci. Rep..

[11] Lahiri B.B., Bagavathiappan S., Jayakumar T., Philip J. (2012). Medical applications of infrared thermography: A review. Infrared Phys. Technol..

[12] Pulia M.S., Schwei R.J., Alexandridis R., Lasarev M.R., Harwick E., Glinert R., Haleem A., Hess J., Keenan T.D., McBride J.A. (2024). Validation of Thermal Imaging and the ALT-70 Prediction Model to Differentiate Cellulitis From Pseudocellulitis. JAMA Dermatol..

[13] Raff A.B., Ortega-Martinez A., Chand S., Rrapi R., Thomas C., Ko L.N., Garza-Mayers A.C., Dobry A.S., Parry B.A., Anderson R.R. (2021). Diffuse Reflectance Spectroscopy with Infrared Thermography for Accurate Prediction of Cellulitis. JID Innov..

[14] Nkengne A., Papillon A., Bertin C. (2012). Evaluation of the cellulite using a thermal infra-red camera. Skin Res. Technol..

[15] Sá D., Mendonça M.I., Sousa F., Abreu G., Ferreira M., Henriques E., Freitas S., Rodrigues M., Borges S., Guerra G. (2025). Association of Obesity-Related Genetic Variants with Android Fat Patterning and Cardiometabolic Risk in Women. Genes.

[16] Wilczyński S., Koprowski R., Deda A., Janiczek M., Kuleczka N., Błońska-Fajfrowska B. (2017). Thermographic mapping of the skin surface in biometric evaluation of cellulite treatment effectiveness. Skin Res. Technol..

[17] Iacobini C., Vitale M., Haxhi J., Menini S., Pugliese G. (2024). Impaired Remodeling of White Adipose Tissue in Obesity and Aging: From Defective Adipogenesis to Adipose Organ Dysfunction. Cells.

[18] Dahdah N., Tercero-Alcázar C., Malagón M.M., Garcia-Roves P.M., Guzmán-Ruiz R. (2024). Interrelation of adipose tissue macrophages and fibrosis in obesity. Biochem. Pharmacol..

[19] Li S., Gao H., Hasegawa Y., Lu X. (2021). Fight against fibrosis in adipose tissue remodeling. Am. J. Physiol. Endocrinol. Metab..

[20] Li Q., Spalding K.L. (2022). Profiling hypertrophic adipocytes in humans, from transcriptomics to diagnostics. eBioMedicine.

[21] De Sousa Neto I.V., Durigan J.L.Q., da Silva A.S.R., de Cássia Marqueti R. (2022). Adipose Tissue Extracellular Matrix Remodeling in Response to Dietary Patterns and Exercise: Molecular Landscape, Mechanistic Insights, and Therapeutic Approaches. Biology.

[22] Liu F., He J., Wang H., Zhu D., Bi Y. (2020). Adipose Morphology: A Critical Factor in Regulation of Human Metabolic Diseases and Adipose Tissue Dysfunction. Obes. Surg..

[23] Mitchell D., Maloney S.K., Snelling E.P., Hetem R.S., Fuller A. (2025). Revisiting Concepts of Thermal Physiology: Understanding Feedback and Feedforward Control, and Local Temperature Regulation. Acta Physiol..

[24] Mazurkiewicz J., Bauer J., Mosion M., Migasiewicz A., Podbielska H. (2018). Severity of Cellulite Classification Based on Tissue Thermal Imagining.

[25] Sun B., Wu J., Li C., Shen S., Han X., Hu Z. (2025). Skin temperature indices to evaluate thermal perceptions and cognitive performance in cold environments. Energy Build..

[26] Lubkowska A., Knyszyńska A. (2023). Thermographic Assessment of Skin Temperature Changes following Partial Body Cryostimulation (PBC) in Football Players. Appl. Sci..

[27] Shinkawa M., Mugita Y., Takahashi T., Haba D., Sanada H., Nakagami G. (2025). A novel skin temperature estimation system for predicting pressure injury occurrence based on continuous body sensor data: A pilot study. Clin. Biomech..

[28] Sadick N. (2019). Treatment for cellulite. Int. J. Women’s Dermatol..

[29] Arora G., Patil A., Hooshanginezhad Z., Fritz K., Salavastru C., Kassir M., Goldman M.P., Gold M.H., Adatto M., Grabbe S. (2022). Cellulite: Presentation and management. J. Cosmet. Dermatol..

[30] LaTowsky B., Jacob C., Hibler B.P., Lorenc P.Z., Petraki C., Palm M. (2023). Cellulite: Current Treatments, New Technology, and Clinical Management. Dermatol. Surg..

[31] Piotrowska A., Czerwińska-Ledwig O., Stefańska M., Pałka T., Maciejczyk M., Bujas P., Bawelski M., Ridan T., Żychowska M., Sadowska-Krępa E. (2022). Changes in Skin Microcirculation Resulting from Vibration Therapy in Women with Cellulite. Int. J. Env. Res. Public Health.

[32] Castellanos-García I., Ramírez-Zuluaga L., Páez-Cárdenas L., Villalba-Moreno D., Caicedo-León M., Singer E., Pincelli T., Bruce A., Aristizabal-Torres M. (2024). Cellulite & Skin Tightening: A Review of Pathophysiology and Topical Treatment. Dermatol. Rev..

[33] Kruglikov I.L., Scherer P.E. (2023). Pathophysiology of cellulite: Possible involvement of selective endotoxemia. Obes. Rev..

[34] Aksu U., Yavuz-Aksu B., Goswami N. (2024). Microcirculation: Current Perspective in Diagnostics, Imaging, and Clinical Applications. J. Clin. Med..

