# Supplementary material for: Validation of Infrared Thermal Imaging for Grading of Cellulite Severity: Correlation with Clinical and Anthropometric Assessments
Source: J Clin Med. 2026 Jan 22;15(2):913. doi: 10.3390/jcm15020913 (PMC12842189; doi:10.3390/jcm15020913)
Supplement: Supplementary file 1 [file jcm-15-00913-s001.zip › jcm-3983403-supplementary.pdf]

Supplementary Table S1: The table presents the Thermographic Cellulite Severity Rating Scale.

Degree of cellulite	Temperature deviation	Skin appearance	Skin color	Skin view	
0	The difference in temp. max.. from min.. from 0.5 to 1.9°C	No change in the appearance of the skin, equal warming skin, smooth skin, no depressions, surface with uniform color	Skin colors in shades of: blue and green	Uniform color, no visible changes	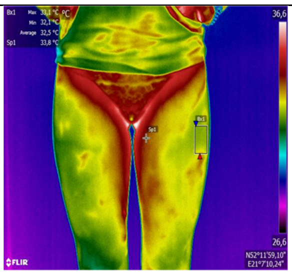
I	The difference in temp. max. from min. from 2.0 to 2.9°C	There are visible areas of higher congestion surrounded by regions with ischemia, which illustrates the non-uniform appearance of the skin, noticeable slight changes in skin temperature, and the skin has become less elastic, showing slight visible color changes. The image is not uniform	Skin colors in shades: Blue, green, and yellow.	There are minimal changes in yellow-green dots, dots, and spots on a blue or green background. Small lesions, clearly demarcated colors.	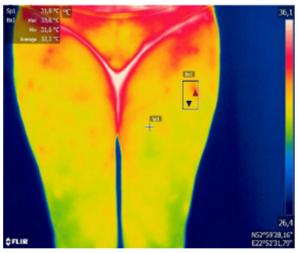
II	The difference in temp. Max.. from min. 3.0 to 3.9°C.	Large areas of congestion, indistinctly demarcated from areas of ischemia, are visible. Slightly more significant changes in skin temperature can be noticed. The skin's appearance is somewhat altered, appearing pale and less elastic.	Skin colors in shades: blue, green, yellow. The dominant colors are blue and green.	On a blue background, green-yellow lesions in the form of small "dots".	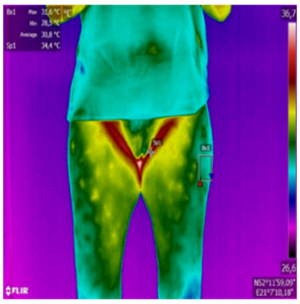
III	The difference in temp. max. from min. from 4.0 to 4.9°C	Large areas of ischemia can be seen, giving the appearance of the so-called "leopard skin," noticeable changes in skin warming, small lumps are visible, and changes in the skin in the form of demarcated "leopard spots."	Skin colors in shades: blue, green, yellow, red. The dominant colors are yellow and red.	The blue background shows green-yellow lesions with red spots and mottling. The lesions are visible and diffuse, quite demarcated.	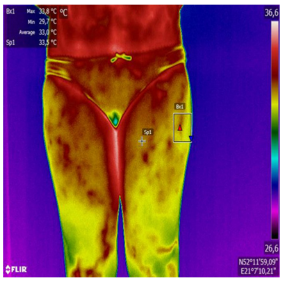

Degree of cellulite	Temperature deviation	Skin appearance	Skin color	Skin view	
IV	Difference in max. Temp. from min. from 5.0 to 5.9°C	Large areas of ischemia can be seen, which give the appearance of the so-called "leopard skin" and the so-called "black hole" area. Evident changes in skin warming, larger lumps are visible, and changes in the skin are in the form of depressions of the so-called "black holes."	Skin colors are in shades of yellow and red.<br>Yellow and red are the dominant colors, but to a much more intense degree than in grade III.	The yellow background features clearly demarcated red lesions in spots, mottles, and holes, creating high contrast between colors. Evident changes.	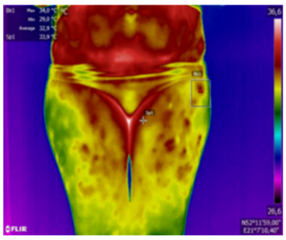
